# Choosing a growth chart for preterm infants: does it matter?

**DOI:** 10.1016/j.jped.2026.101614

**Published:** 2026-09-17

**Authors:** Stephanie Merlino Barr

**Affiliations:** MetroHealth Medical Center, Cleveland, OH, USA

In this edition of *Jornal de Pediatria,* the work of da Cunha, et al, evaluates the implications of growth chart selection for growth analysis in the preterm infant population [1]. This work compares birthweight z-score and birth size categorization between the Fenton 2013 and INTERGROWTH preterm infant growth charts [2,3]. While the presented Bland Altman analyses show generalized agreement between the two growth charts, a pattern of poor agreement was evident among more premature and smaller infants. This analysis expertly demonstrates that the two growth charts are not interchangeable, and differences between them could be clinically meaningful. Two important questions arise from this work: how will the recently published Fenton 2025 growth charts compare to the INTERGROWTH charts, and which growth chart should be used in a clinical neonatal intensive care unit (NICU) setting?

The publication of the updated Fenton 2025 charts necessitates renewed examination of whether commonly used preterm growth references provide equivalent assessments of birth size, particularly at the extremes of gestational age and birth weight [1,4]. A separate, previously published dataset was utilized to assess these questions, which included all infants admitted to a level III NICU between 2013 and 2022 and excluded those who died during their initial NICU course and those who had a length of stay less than 14 days, to match the inclusion and exclusion criteria of da Cunha, et al.’s work [5]. A total of 1575 infants were included in this analysis, with an average birth gestational age of 31.8 weeks (SD = 3.5) and mean birthweight of 1694 grams (SD = 657). Birthweight z-scores were assigned using the Fenton 2013 (https://peditools.org/peditools_universal/), Fenton 2025 (https://fentongrowth.ca/), and INTERGROWTH (https://intergrowth21.ndog.ox.ac.uk/preterm) growth charts, using available online calculators [[2], [3], [4],^6^].

This re-analysis found a similar dispersion of birth size status of this study population using the Fenton 2013 and INTERGROWTH charts as compared to da Cunha, et al.’s analysis; the Fenton 2025 had a larger proportion of infants categorized as small for gestational age (SGA, birthweight < 10^th^ percentile) and large for gestational age (LGA, birthweight > 90^th^ percentile) compared to average for gestational age (AGA, birthweight between 10 – 90^th^ percentile) (Table 1). Bland Altman graphs show comparisons between the Fenton 2013 and Fenton 2025 (Fig. 1A), Fenton 2013 and INTERGROWTH (Fig. 1B), and Fenton 2025 and INTERGROWTH charts (Fig. 1C) with full summary statistics presented in Table 2. Comparisons of the Fenton 2013 and Fenton 2025 charts show expected moderate to poor agreement, given the changes in inclusion criteria in the updated charts (Fig. 1A). The comparison of Fenton 2013 and INTERGROWTH show similar trends as described by da Cunha, et al., with fair overall agreement, but less consistent agreement in SGA and more premature infants (Fig. 1B). The comparison between Fenton 2025 and INTERGROWTH shows a distinctly poorer pattern of agreement (Fig. 1C), with a large mean bias overall and across all prematurity categories. These findings support what da Cunha, et al. report, which is that preterm infant growth charts are not interchangeable, and may have differing clinical implications.

**Table 1:** Birth size categorizations by each growth chart.

	Fenton 2013	Fenton 2025	INTERGROWTH
n	1570	1570	1527
n excluded due to growth chart age limitations	5	5	48
SGA	195 (12.4%)	380 (24.2%)	243 (15.9%)
AGA	1307 (83.3%)	1090 (69.4%)	1208 (79.1%)
LGA	68 (4.3%)	100 (6.4%)	76 (5.0%)

**Figure 1:**
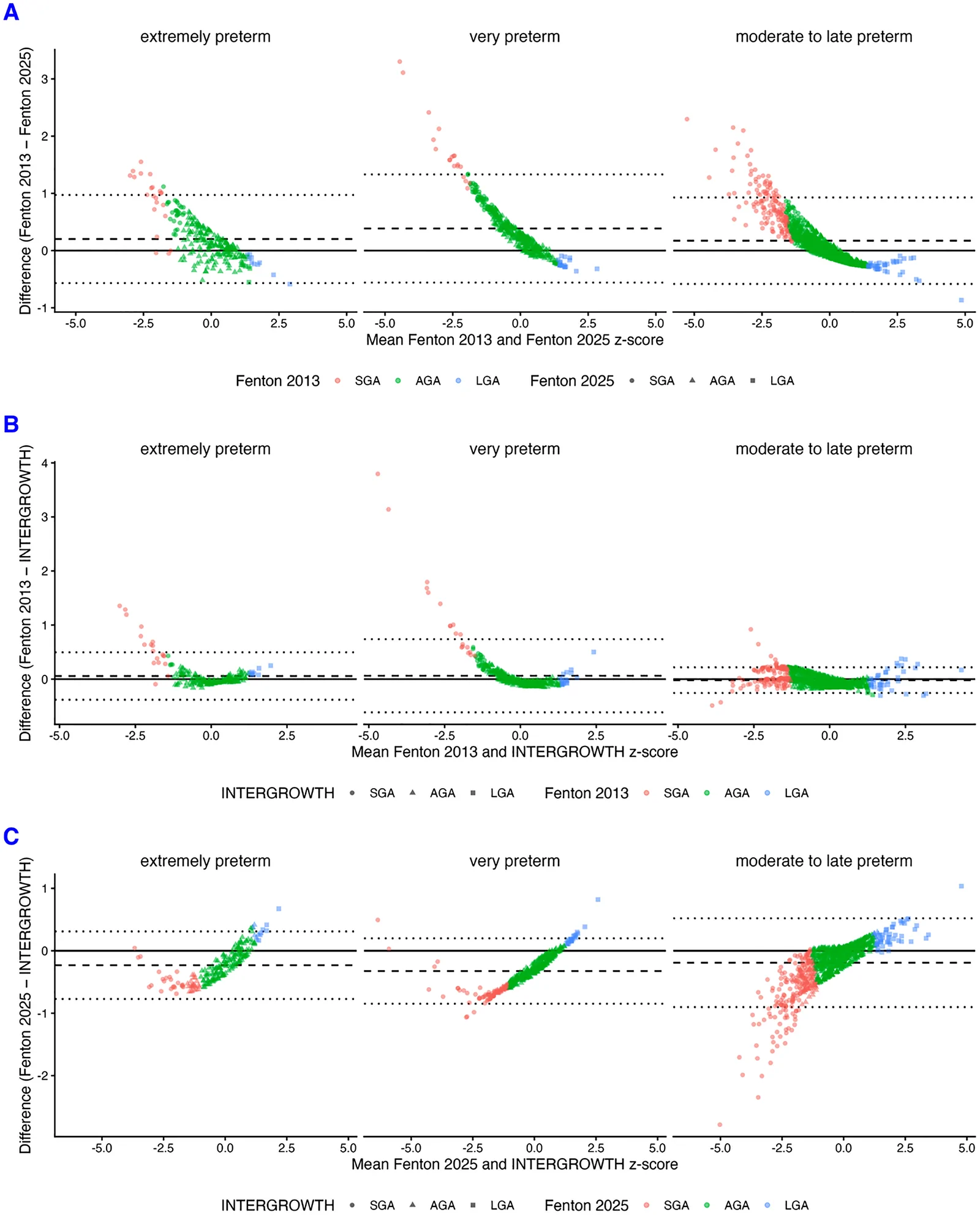
Bland Altman Analysis of Fenton 2013, Fenton 2025, and INTERGROWTH growth charts.

**Table 2:** Bland-Altman analyses comparing Fenton 2013, Fenton 2025, and INTERGROWTH weight-for-age z-scores at birth.

	Mean Bias	SD	-2SD	+2SD
*Fenton 2013 vs Fenton 2025*				
Overall	0.23	0.42	-0.60	1.06
Extremely Preterm	0.20	0.39	-0.57	0.98
Very Preterm	0.39	0.48	-0.56	1.33
Moderate to Late Preterm	0.17	0.39	-0.58	0.93
*Fenton 2013 vs INTERGROWTH*				
Overall	0.01	0.22	-0.42	0.44
Extremely Preterm	0.06	0.22	-0.38	0.50
Very Preterm	0.06	0.35	-0.61	0.74
Moderate to Late Preterm	-0.02	0.12	-0.26	0.22
*Fenton 2025 vs INTERGROWTH*				
Overall	-0.23	0.33	-0.89	0.42
Extremely Preterm	-0.23	0.28	-0.77	0.31
Very Preterm	-0.32	0.27	-0.85	0.20
Moderate to Late Preterm	-0.19	0.36	-0.90	0.52

With differences among growth references, what should guide the clinical selection of a growth chart? Understanding the differences in data sources (see Table 3), as well as the limitations of available growth assessment tools, is essential to making an informed clinical decision. The Fenton 2013 growth charts are a meta-analysis of cross-sectional data showing size-at-birth of preterm infants from six country-specific data sources [2]. The Fenton 2013 growth chart incorporates the WHO growth standards extending the range of the growth chart to 50 weeks postmenstrual age (PMA) [2]. The Fenton 2013 growth chart is commonly used in clinical practice due to the 22 to 50 week PMA range available for monitoring infant growth. However, the Fenton 2013 growth chart does have some important limitations, namely, it does not necessarily describe ideal fetal growth, as infants born extremely prematurely are often not representative of a healthy pregnancy or fetal environment. The INTERGROWTH growth charts describe growth standards, developed by utilizing longitudinal growth data measured using best practices, recruiting infants from low-risk pregnancies without evidence of intrauterine growth restriction, and recruited from NICUs utilizing prescribed nutrition best practices [3]. While the INTERGROWTH charts address some of the limitations of the Fenton 2013 charts, they start at 27 weeks PMA and include data from only 4 infants born less than 30 weeks PMA in their development, making it a limiting tool in the growth evaluation of extremely preterm infants. The Fenton 2025 growth charts were developed with a similar methodology as the 2013 charts but limited included birth data to infants without abnormal fetal growth by excluding those with perinatal morbidities (e.g., congenital anomalies, hypertension, maternal smoking) and provider-initiated births. These methodological changes address limitations of the 2013 charts to better describe ideal fetal growth while also supporting growth assessment of preterm infants beginning at 22 weeks PMA.

**Table 3:** Comparison of growth chart composition.

	Fenton, 2013#	Fenton, 2025%	INTERGROWTH$
Dates	1991 – 2007	1996 – 2021	2009 – 2014
Sample size	3,986,456	4,929,075	201
N < 30 weeks	34,639	174,184	4
Lowest gestational age, weeks	22	22	27
Method of data collection	Cross-sectional	Cross-sectional	Longitudinal

# Data from: Fenton TR, Kim JH. 2013. doi:10.1186/1471-2431-13-59

% Data from: Fenton TR, Elmrayed S, Alshaikh BN. 2025. doi:10.1111/ppe.70035

$ Data from: Villar J, et al. 2015. doi:10.1016/S2214-109X(15)00163-1.

The American Academy of Pediatrics recommends that preterm infants should approximately grow at the rate and composition of a fetus [7]. Growth of preterm infants may differ from fetal growth due to diuresis post birth, but should approximately follow fetal growth patterns after initial diuresis [8,9]. There is no agreed upon gold standard of preterm infant growth chart, although the INTERGROWTH charts do propose preterm infant growth standards and the Fenton 2025 charts seek to describe a healthier birth population. From a clinical perspective, implementation of a single tool that can be used for the majority of NICU patients may make the Fenton 2025 charts a pragmatic choice for routine clinical use. As our understanding of preterm infant growth continues to evolve, our available tools to assess growth will continue to change. As demonstrated by da Cunha, et al., growth charts should not be used interchangeably. Growth references are not just descriptive tools; selection of growth charts will influence birth size classification, research populations, and estimates of growth-related outcomes.

## Conflicts of interest

Dr. Merlino Barr has received honoraria for the creation of educational materials and presentations for Abbott Nutrition Health Institute, AVANOS, Baxter Healthcare, Nutricia, and Reckitt/Mead Johnson.
